# The Temporal Transcriptomic Response of *Pinus massoniana* Seedlings to Phosphorus Deficiency

**DOI:** 10.1371/journal.pone.0105068

**Published:** 2014-08-28

**Authors:** Fuhua Fan, Bowen Cui, Ting Zhang, Guang Qiao, Guijie Ding, Xiaopeng Wen

**Affiliations:** 1 Key Laboratory of Plant Resources Conservation and Germplasm Innovation in Mountainous region (Guizhou University), Ministry of Education, Institute of Agro-bioengineering, Guizhou University, Guiyang, Guizhou Province, People’s Republic of China; 2 School of Forestry Science, Guizhou University, Guiyang, Guizhou Province, People’s Republic of China; 3 The School of Nuclear Technology and Chemical and Biological, Hubei University of Science and Technology, Xianning, Hubei Province, People’s Republic of China; National Institute of Genomic Medicine, Mexico

## Abstract

**Background:**

Phosphorus (P) is an essential macronutrient for plant growth and development. Several genes involved in phosphorus deficiency stress have been identified in various plant species. However, a whole genome understanding of the molecular mechanisms involved in plant adaptations to low P remains elusive, and there is particularly little information on the genetic basis of these acclimations in coniferous trees. Masson pine (*Pinus massoniana*) is grown mainly in the tropical and subtropical regions in China, many of which are severely lacking in inorganic phosphate (Pi). In previous work, we described an elite *P. massoniana* genotype demonstrating a high tolerance to Pi-deficiency.

**Methodology/Principal Findings:**

To further investigate the mechanism of tolerance to low P, RNA-seq was performed to give an idea of extent of expression from the two mixed libraries, and microarray whose probes were designed based on the unigenes obtained from RNA-seq was used to elucidate the global gene expression profiles for the long-term phosphorus starvation. A total of 70,896 unigenes with lengths ranging from 201 to 20,490 bp were assembled from 112,108,862 high quality reads derived from RNA-Seq libraries. We identified 1,396 and 943 transcripts that were differentially regulated (P<0.05) under P1 (0.01 mM P) and P2 (0.06 mM P) Pi-deficiency conditions, respectively. Numerous transcripts were consistently differentially regulated under Pi deficiency stress, many of which were also up- or down-regulated in other species under the corresponding conditions, and are therefore ideal candidates for monitoring the P status of plants. The results also demonstrated the impact of different Pi starvation levels on global gene expression in Masson pine.

**Conclusions/Significance:**

To our knowledge, this work provides the first insight into the molecular mechanisms involved in acclimation to long-term Pi starvation and different Pi availability levels in coniferous trees.

## Introduction

Phosphorus (P) is an essential mineral macronutrient that is crucial for plant development and metabolism. Plants acquire P in the form of inorganic phosphate (Pi) through their roots from water in the soil. Although Pi is generally abundant in soils, the availability for plant uptake is often limited due to high fixation and slow diffusion [1], [2], making P one of the major factors that constrain plant growth and productivity worldwide [2], [3]. Economically important crops and forestry plantations are therefore frequently supplemented with Pi fertilizer to maximize yields and quality. Unfortunately, the application of Pi fertilizer can cause severe environmental damage, such as eutrophication of water systems [4]. Pi fertilizer use also significantly increases the cost of crop production, particularly for forest cultivation, and worldwide Pi resources will be exhausted in the near future if the current rate of extraction continues [3], [4]. Thus, a fuller understanding of the strategies used by plants to uptake and utilize Pi is of high importance and may be applied to the breeding or engineering of plants with greater capacity to acquire, transport, store, and recycle soil Pi [5]–[7]. Plants have evolved a variety of adaptive strategies to cope with Pi-deficiency that involve in morphological, physiological, biochemical, and molecular responses. For example, plants may modify root architecture and morphology [8], [9], increase the exudation of H^+^, organic acids and phosphatases from roots [10]–[12], optimize internal Pi use [13], and elevate the expression of high-affinity phosphate transporters [14]–[16].

It is believed that these adaptive strategies are mediated by the coordinated function of hundreds of plant genes. Over the past two decades, extensive studies on the response to Pi starvation in the model plant *Arabidopsis thaliana* have contributed significantly to our understanding of P signaling and response pathways [17]–[19]. Meanwhile, transcriptional profiling of *A. thaliana* [20] and other important crop plants such as *Oryza saliva* (rice) [21], *Zea mays* (maize) [22], *Brassica rapa* (Chinese cabbage) [23], *Cucumis melo* (melon) [24], *Phaseolus vulgaris* (common bean) [25] and *Lupinus albus* (white lupin) [16] have provided knowledge at the whole genome scale and revealed the complexity of the network of genes necessary for plants to adapt to low soil Pi availability. A series of genes confer adaptation to Pi-deficiency, mainly through the regulation of P acquisition, internal remobilization, changes in metabolism, and signal transduction [26]. Expression of genes encoding purple acid phosphatases (PAPs) and Pi transporters were found to be generally up-regulated under Pi starvation, and these proteins are important for mobilization of soil organic P for root absorption and transportation [27], [28]. Pi-deficiency also up-regulated several genes involved in lipid metabolism, including nonspecific phospholipase and UDP sulfoquinovose synthases [29], and affected carbon metabolism [13]. Numerous regulatory genes involved in response to Pi-deficiency have also been identified, which included transcription factors (*TFs*), sugar-phosphate exchanger subfamily proteins (*SPX*, *SYG1*, *Pho81*, *XPR1*), plant hormones and protein modifiers, as well as noncoding RNAs [30]–[32].

Masson pine (*Pinus massoniana*), a gymnosperm belonging to the conifer genus, is native to southern China and is one of the most economically important forest trees that is widely used for timber, pulp and resin production. This tree species is grown mainly in tropical and subtropical regions, many of which are severely stricken with Pi-deficient soils, which constrains the productivity of this and other forest crops [33]. Several researches had been carried out to improve the P efficiency [34]–[37], however, the genes involved in the Pi starvation response in Masson pine have not yet been unraveled to date.

Gene expression microarray analysis may generate a wealth of important information on transcriptional changes in response to different stimuli, and have contributed enormously to many areas of the life sciences [38]. Nevertheless, microarray methods suffer some potential pitfalls, and RNA-seq approaches can provide superior reproducibility and accuracy, and can reveal transcriptional changes over a wider dynamic range. Thus, RNA-seq techniques are increasingly used to accurately quantify gene expression levels [39]–[42]. Kogenaru et al. (2012) showed that RNA-seq and microarray approaches can mutually complement demerits, and their combination may provide a more comprehensive understanding of the transcriptome [43].

An elite Masson pine germplasm, which showed superior tolerance to Pi-deficiency, has been obtained from our previous work. In order to investigate the complex molecular mechanisms involved, transcriptional profiling was performed by a combination of RNA-seq and microarray methodologies in this germplasm under Pi-sufficient and Pi-deficient conditions. The effects of the magnitude and duration of Pi-deficient conditions on global gene expression were investigated. The results provided insight into the molecular mechanisms involved in acclimation to long-term Pi starvation at different levels of Pi-deficiency in coniferous trees, and this knowledge may be applied to other plants. The differentially expressed genes identified in this study will facilitate the further genomic and genetic engineering studies on Masson pine and other important crops.

## Materials and Methods

### Plant materials and growth conditions

Masson pine seeds were collected from a first-generation orchard located in the Meishan Forest Farm of Sichuan Province (P. R. China) (E: 103.21; N: 29.25). These seeds were shown to have a high ability to survive in Pi-deficient conditions in our previous work. Field studies did not involve endangered or protected species, and no specific permissions were required for this location and activities. Seeds were surface sterilized, rinsed in sterile water, and allowed to imbibe overnight in a tray containing warm sterile water at 30°C. Sterilized seeds were planted for six seedlings per 12 centimetres diameter pot containing quartz sand. Pots were placed in growth chambers at 25°C/16°C with a 14-h photoperiod (2000Lx), and watered once per day since 10 days after emergence (DAE). Three treatments were carried out with different P levels: i) control nutrient solution containing 5.0 mM KNO_3_, 4.5 mM Ca(NO_3_)_2_·4H_2_O, 2.0 mM MgSO_4_·7H_2_O, 0.5 mM KH_2_PO_4_, 46 µM H_3_BO_3_, 10 µM MnCl_2_·4H_2_O, 0.8 µM ZnSO_4_·7H_2_O, 0.56 µM CuSO_4_·5H_2_O, 0.4 µM H_2_MoO_4_·4H_2_O, and 25 µM Fe-NaEDTA; ii) nutrient solution deficient in Pi (P1) containing 0.01 mM KH_2_PO_4_; iii) nutrient solution deficient in Pi (P2) containing 0.06 mM KH_2_PO_4_. KCl was added to Pi-deficient solutions to ensure identical potassium concentrations. Ten-day-old seedlings were divided into three groups, which were grown under one of the three P levels. The nutrient solution was added at a rate of 100 mL per pot every two days. Seedlings were harvested 22, 34, 58 and 70 DAE, immediately frozen in liquid nitrogen and stored at −80°C for RNA extraction. All treatments were replicated in three pots.

### RNA isolation, cDNA library construction and sequencing

Total RNA was extracted from the samples using an Invitrogen Plant RNA Isolation Kit and purified using an RNeasy MiniElute Cleanup Kit (Qiagen), both according to the manufacturers’ protocols. RNA was quantified using a Qubit RNA Assay Kit (Invitrogen), and RNA integrity was checked with the RNA6000 Nano Assay using an Agilent 2100 Bioanalyzer (Agilent Technologies). RNA isolated from Pi-deficient samples (P1 and P2) and Pi-sufficient (control) samples were mixed separately for library preparation and sequencing. cDNA library preparation and deep sequencing were conducted in the Biochip National Engineering Research Center of Beijing, CapitalBio Corporation. For Illumina library construction and sequencing, RNA samples were prepared using a TruSeq RNA Sample Preparation Kit (Illumina) according to the manufacturer’s recommendations. The library was qualified using an Agilent 2100 Bioanalyzer and quantified by Qubit and qPCR assays. Cluster formation and sequencing were performed on a HiSeq2000 platform (Illumina) following the manufacturer’s standard cBot and sequencing protocols. Primary data analysis and base calling were performed using the Illumina instrument software.

### Transcriptome assembly, functional annotation and classification

To generate a non-redundant set of transcripts, we performed an assembly with publicly available programs using 112,108,862 RNA-seq reads obtained from two libraries. High quality statistics were selected on the basis of Q20. The reads were assembled using Trinity software (Grabherr et al., 2011) to construct unique consensus sequences. Unigenes were compared with the NCBI non-redundant nucleotide database (Nt, January, 2013; http://www.ncbi.nlm.nih.gov/) and non-redundant protein database (Nr, January, 2013; http://www.ncbi.nlm.nih.gov/) using BLASTN and BLASTX, respectively, with E-value cutoffs≤1e-5. Putative functions were assigned to unique sequences by sequence similarity comparison against the Clusters of Orthologous Groups of proteins (COG) database [44], [45] with BLAST at E values≤1e-10. InterPro domains were annotated using InterProScan Release 27.0 and functional assignments were mapped onto the Gene Ontology (GO) entries [46]–[48]. Assembly sequence data was submitted to the NCBI Transcriptome Shotgun Assembly (TSA) database (accession number GAQR00000000).

### Microarray preparation and data analysis

Unique 60-mer oligonucleotide probes were designed for each of the >56,000 high quality unigenes identified in this work. 8×60-K DNA microarray chips were implemented with the Agilent platform. A total of 12 sample RNAs consisting of one of each of the three different P levels from each experimental duration were further purified using an RNA Cleanup Kit (Macherey-Nagel, Germany). Total RNA was used with a cDNA Amplification Tag Kit (Jingxin, CapitalBio Corporation, China) and a NucleoSpinExtract II Kit (MN) to generate the targets. Each mRNA sample was reverse-transcribed in the presence of Cy3-dUTP or Cy5-dUTP (GE Healthcare) and 5 µg of labeled cDNA was hybridized with the microarray. After washing, microarray plates were dried briefly and scanned with an Agilent G2565CA Microarray Scanner. The ratios of the two fluorescent signal intensities of each DNA element were then measured to determine changes in gene expression. Microarray data were processed at the Biochip National Engineering Research Center of Beijing, CapitalBio Corporation. Analysis was performed using Feature Extraction and GeneSpring GX software with RMA normalization. Genes were considered differentially expressed if up-regulated by >2-fold or down-regulated by <0.5-fold (P<0.05). Microarray data have been deposited in the Gene Expression Omnibus database (accession number GSE52835).

### Quantitative real-time PCR verification

Total RNA was isolated from seedlings grown under different Pi treatments 22, 34, 58 and 70 DAE as described above. First-strand cDNA was synthesized using an RNA LA PCR Kit (TaKaRa, Dalian, China) with the supplied oligo dT-adaptor primer following the manufacturer’s instructions. Gene-specific primers (Table 1) were designed for nine randomly selected unigenes involved in Pi-deficiency using Primer Premier 5.0. Samples and standards were performed in triplicate on each plate using the SYBR Select Master Mix Kit (Applied Biosystems) on a 7500 fast real-time PCR system (Applied Biosystems) following the manufacturer’s instructions. Quantitative real-time PCR (qRT-PCR) was performed in a 20 µL reaction containing 7.5 µL of deionized water, 10 µL of SYBR mix, 0.5 µL of forward primer (10 µM), 0.5 µL of reverse primer (10 µM), and 1.5 µL of template cDNA. The PCR procedure was as follows: 2 min of pre-denaturation at 95°C; 40 cycles of 20 s at 95°C, 25 s at 58°C, and 60 s at 68°C; and an additional step for dissociation (15 s at 95°C, 60 s at 60°C, and 15 s at 95°C). The 7000 System SDS Software (Applied Biosystems) was used for data collection. Relative transcript levels for each sample were determined using the comparative cycle threshold (Ct), and normalized against the geometric mean of the Ct value of three internal control genes (18s RNA; ubiquitin-conjugating enzyme-like protein, UBC; NAD-dependent glyceraldehyde-3-phosphate dehydrogenase, GAPDH). Expression ratios were calculated using the ΔΔCt method corrected for the PCR efficiency for each gene.

**Table 1:** Primers used for the quantitative real-time assay.

Transcript No.	Putative identity	Primer sequence
**comp24826_c0_seq2**	Lipid transport	F: 5′-TCCAAGGTGGTTAGCG-3′
		R: 5′-CAAAGTCAACCGCAAT-3′
**comp31360_c0_seq5**	Peptide transporter ptr1-like	F: 5′-AAATACTGCCTTCTTGAC-3′
		R: 5′-TTTACCGACATAAACTGC-3′
**comp16900_c1_seq1**	Myb superfamily proteins	F: 5′-GTAAGGTTTCTGCGTTCA-3′
		R: 5′-CCTTGGCTAAAGCACTCC-3′
**comp69613_c0_seq1**	WRKY transcription factor	F: 5′-CGCCATAGCACATCTGTA-3′
		R: 5′-AGAGGCTCAGCAACGACT-3′
**comp30649_c1_seq2**	Leucine-rich repeat (LRR) protein	F: 5′-TCAACGCCATTCCGATTC-3′
		R: 5′-GCCAGACCAGGAGCATAT-3′
**comp216440_c0_seq1**	Metal ion transport	F: 5′-TCGCACAGACCAGCAGTA-3′
		R: 5′-AGGGCATCACATCACAGAAT-3′
**comp30272_c0_seq3**	Hypothetical protein	F: 5′-AAATGTATTGGCTGATGG-3′
		R: 5′-TAGGGAAACGATAGAAGG-3′
**comp29129_c0_seq3**	PR-4 protein	F: 5′-AGCACAAACGCAGAGCAA-3′
		R: 5′-CCACAGAAGGCAGTCCAT-3′
**comp23145_c2_seq1**	SPX domain-containing membrane protein	F: 5′-CGGGAAATGCTAATGGAG-3′
		R: 5′-TGTTTGAAGACCTGCTGA-3′

## Results

### Construction of the Masson pine transcript dataset

**Figure 1:**
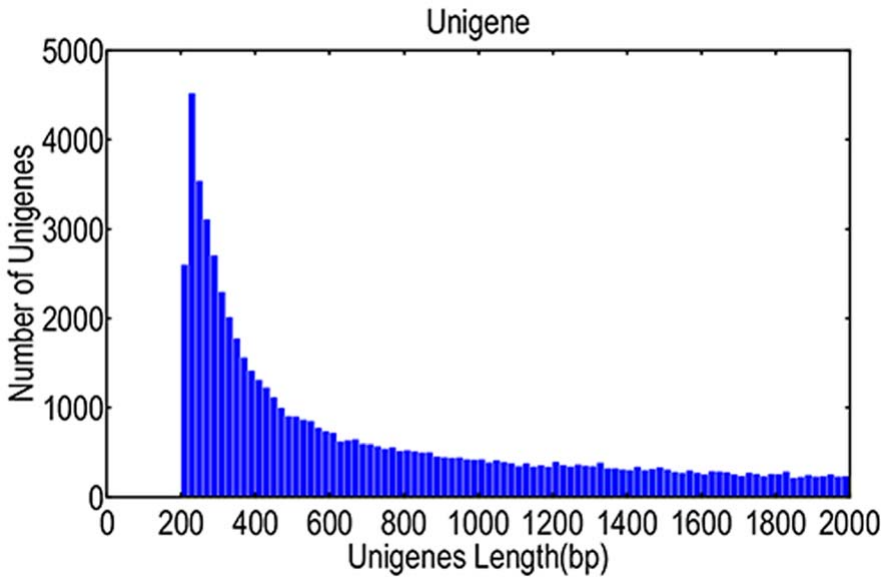
Sequence size distribution and the number of sequences of each length in the assembly (shown on the y-axis).

Even amounts of RNA collected from the seedlings grown under Pi-sufficient (control) or Pi-deficient (P1 and P2) conditions for 12, 24, 48 and 60 days were used to construct two cDNA libraries. Second-generation sequencing was then used to construct the transcript dataset. A total of 70,896 unigenes were assembled from 112,108,862 high quality RNA-seq reads generated from two libraries (as described in the Materials and methods). The length of unigenes ranged from 201 to 20,490 bp, and the total base count of these sequences was 70,169,217 bp (Figure 1).

Unigenes were analyzed for similarity/sequence conservation against non-redundant (nr) datasets of various species using BLAST (E-value ≤1e-5), and over 47,100 unigenes were identified in plants. Putative functions were assigned for 66.3% of the sequences. An additional >56,000 more highly expressed transcripts were manually curated to ensure accurate expression profile analysis of the microarray data.

### Identification of transcripts differentially expressed under Pi-deficient conditions using microarray data

**Figure 2:**
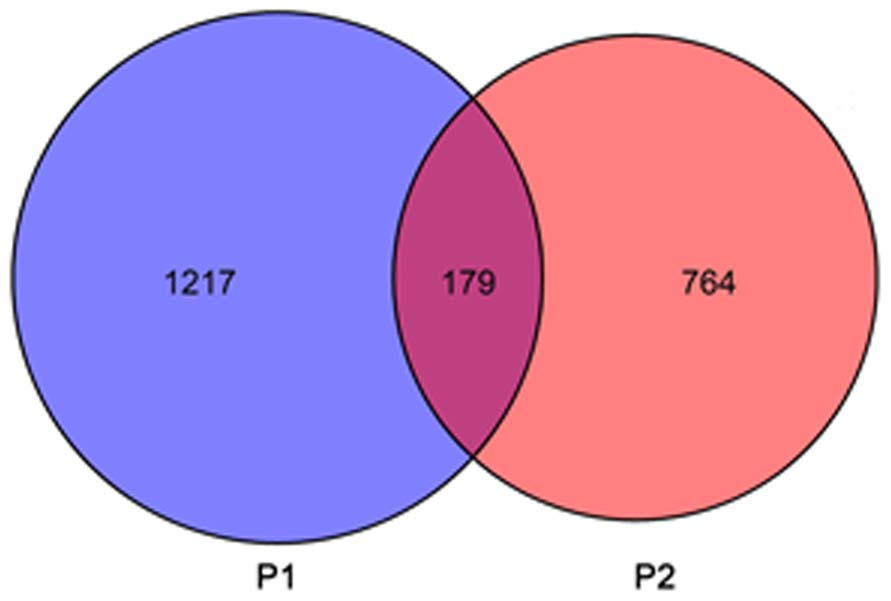
Transcripts differentially expressed under different Pi levels. A total of 2,160 transcripts were identified as differentially expressed under both Pi-deficiency treatments.

**Figure 3:**
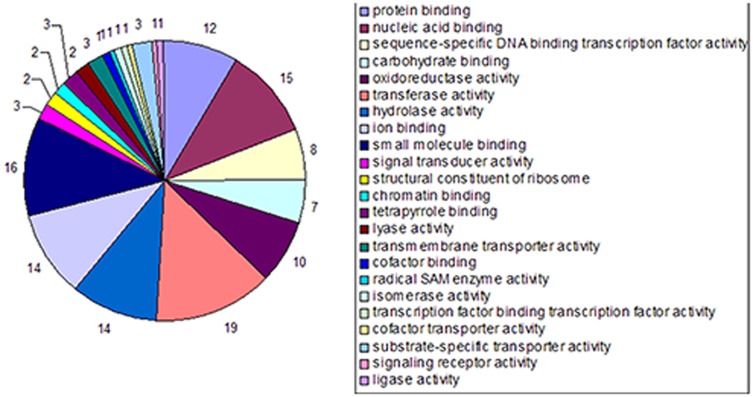
Categorization of 179 transcripts based on molecular function.

A series of microarray experiments were used to measure gene expression in Masson pine seedlings grown under conditions of low Pi for four periods. Using ANOVA with the Benjamini-Hochberg correction for multiple testing, we identified 1,396 and 943 differentially expressed genes (P<0.05) in the four periods under P1 and P2 conditions, respectively (Figure 2; Table S1, S2). Of the 1,396 transcripts identified in P1, 776 were up-regulated, while 544 of the 943 P2 transcripts followed the same trend. The number of transcripts induced in P2 was less than 32% of those induced in P1, suggesting that the growth of Masson pine was more severely limited by lower Pi levels. Analysis of overlap on the Venn diagram (Figure 2) showed that 179 transcripts were induced in P1 and P2. Of these, 14 encoded genes involved in transferring phosphorus-containing groups from donors to acceptors. Another 14 transcripts encoding genes similar to hydrolases that act on esters, glycosyl bonds and acid anhydrides, and a further eight genes encoded sequence-specific DNA binding transcription factors. Other identified genes included several pentatricopeptide repeat-containing proteins, gibberellin induced protein, thaumatin-like protein, and a further 66 cDNAs with unknown function (Figure 3; Table S3).

### Characterization of genes induced by Pi-deficiency

**Figure 4:**
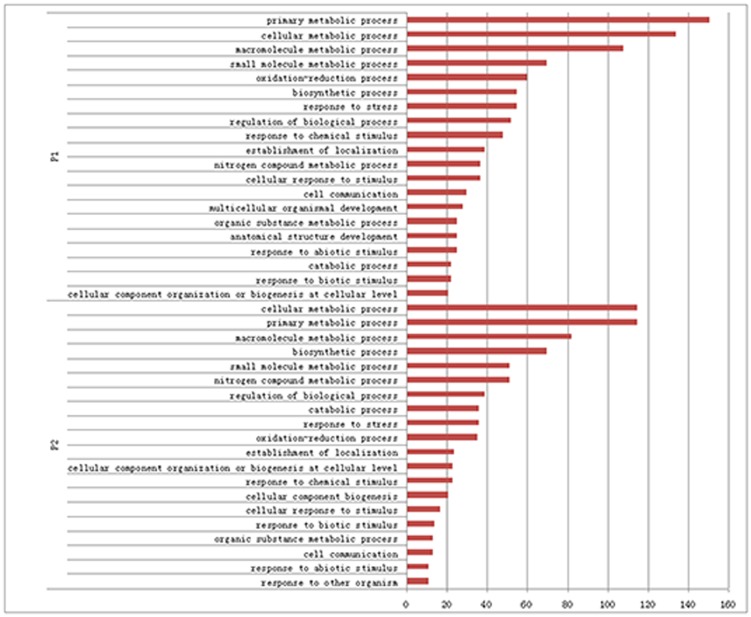
Gene Ontology (GO) categories of up-regulated genes. The numbers of up-regulated transcripts in P1 and P2 seedlings categorized in the top 20 GO groups are shown. The x-axis indicates the number of transcripts in a category, and the y-axis indicates the category.

For functional annotation of transcripts up-regulated under low Pi conditions, Gene Ontology (GO) was used. The top 20 GO categories were identified for P1 and P2 stressed seedlings (Figure 4). Although the numbers of transcripts differed, the overall categorizations were similar between P1 and P2 stressed seedlings, with most involved in metabolic processes, including various transferases, phosphatases, protein kinases, synthases, hydrolases, polymerases and oxidases. Many glycosyltransferases and lipases that involved in lipid metabolism were included, which may alter the membrane lipid composition to maintain intracellular Pi homeostasis under Pi-deficiency [49]. Many P1 and P2 transcripts encoded redox proteins, especially cytochrome P450 enzymes and the 2OG-FeII oxidoreductase, both of which were identified as up-regulated in response to phosphate starvation in *Arabidopsis* and wheat [50], [51]. Other oxidation-reduction-associated protein genes included alcohol dehydrogenase, ascorbate oxidase (AO), laccase, polyphenol oxidase, primary amine oxidase, and flavonoid-associated enzymes were also differentially regulated as subjected to the Pi-deficiency. Most of the remaining differentially expressed transcripts belonged to the stress or stimulus category.

The uptake of Pi from soil alters the expression of transporter proteins (TPs) and TFs, and the expression of these proteins is affected by the Pi abundance. Both TPs and TFs were differentially expressed in P1 and P2 seedlings, and there were differences between the two sets of treatments. A total of 20 different TPs were differentially expressed in P1, while only 10 were differentially expressed in P2 (Table S4). Five important phosphate TPs were only identified in P1 seedlings, and some potassium and drug transmembrane TPs were also only identified in P1 seedlings. Some TP transcripts identified in both P1 and P2 seedlings exhibited significantly different levels; five ABC transporters were highly differentially expressed in P1 while only three were up-regulated in P2, and six nitrate transporters were up-regulated in P1, while only one was present down-regulated in P2. Additionally, 14 TF family proteins were differentially expressed under Pi-deficiency stress conditions (Table S5). AP2, MYB, WRKY and MADS-Box proteins were identified as the major TFs responding to Pi-deficiency stress under both P1 and P2 conditions. Of these, all MADS-Box proteins were down-regulated, whereas, MYB and WRKY TFs were up-regulated. Five GATA TFs and one bHLH TF protein were down-regulated in P1 but not altered in P2.

As noted in previous studies, harmful reactive oxygen species (ROS) can be generated under abiotic stress conditions, including Pi-deficiency [16], [52]. The microarray data obtained in this study revealed up-regulation of glutathione s-transferase (GST, comp20392_c0_seq1) and glutathione peroxidase (comp28364_c0_seq1), and down-regulation of AO (comp25395_c1_seq1, comp13179_c0_seq1, comp12918_c0_seq1, comp12918_c2_seq1) under P1 condition. Additionally, SPX domain-containing genes were also up-regulated in P1 stressed seedlings.

### Expression profiles of Pi-deficiency induced genes

**Figure 5:**
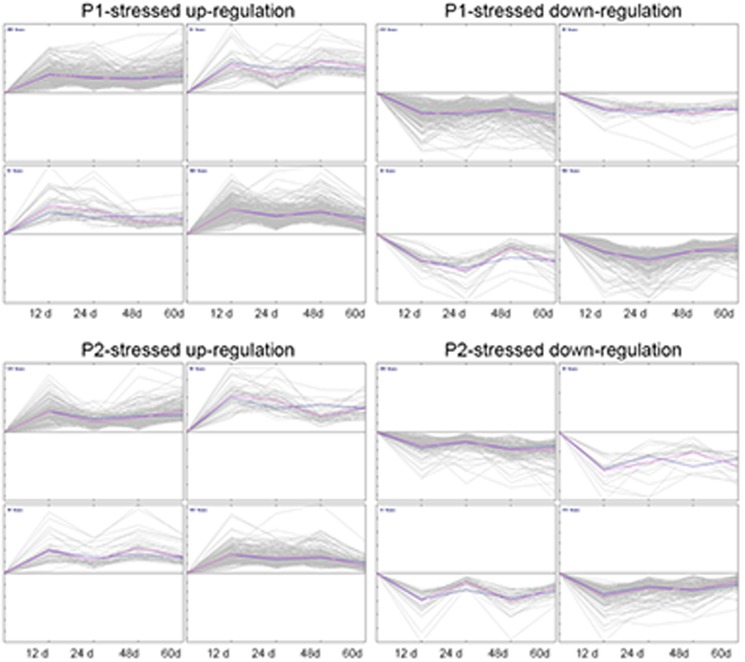
Division of genes differentially expressed by Pi-deficiency into four groups based changes in expression pattern. A time course of changes in expression pattern is shown. Seedlings under P0 conditions are included as a control. The x-axis represents time points after treatment, and the y-axis indicates the expression level.

To obtain insight into the mechanisms involved in the response to Pi-deficiency, transcriptomic analyses were performed on seedlings grown for different durations under the stress conditions. A self-organizing map (SOM) was used for clustering using the GeneSpring software. The expression profiles of differentially expressed transcripts under P1 and P2 treatments were divided into at least four groups (Figure 5), among which most transcripts were stably and gradually up- or down-regulated within 48 days. Interestingly, a number of transcripts increased or decreased much more rapidly and over a wider dynamic range under P1 conditions than did that under P2 conditions (Figure 5).

### Microarray expression validation by qRT-PCR

**Figure 6:**
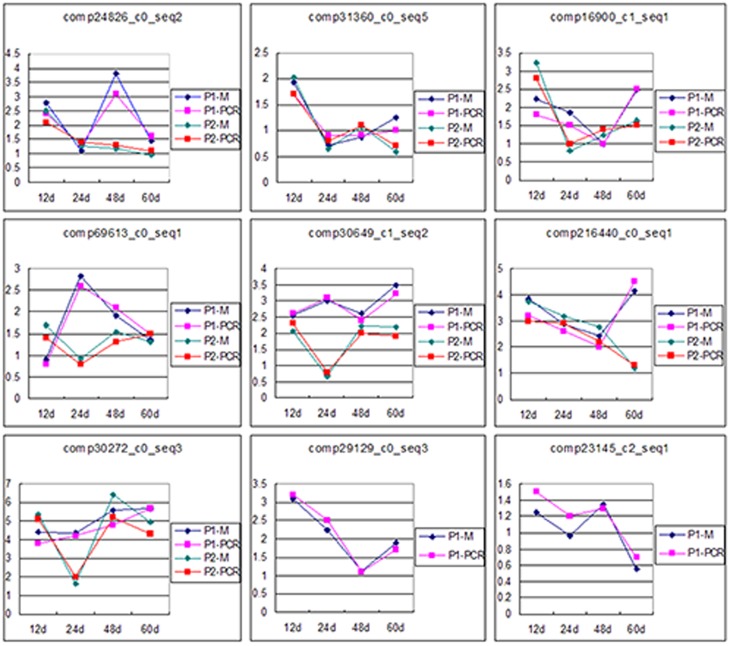
Expression profiles of nine randomly selected transcripts determined by qRT-PCR (-PCR) and microarray (-M). The x-axis indicates the duration of the Pi-deficiency stress, and the y-axis indicates the signal ratio. The conditions of P1 and P2 are described in the Materials and methods.

Generally, qRT-PCR is used to validate gene expression levels determined using high-throughput technologies such as microarray and RNA-seq. Therefore, we further confirmed the microarray expression profiles using qRT-PCR under identical conditions for a set of nine randomly selected unigenes involved in the phosphate starvation response. A high correlation was identified between qRT-PCR and microarray results, confirming the high reliability of the microarray expression data (Figure 6).

## Discussion

Plant acclimation to Pi-deficiency stress is an important topic in plant biology, not least because of our dependence on Pi fertilizers, which are derived from a non-renewable natural resource that is being rapidly depleted. Several studies based on high-throughput technologies have highlighted the highly complex molecular systems involved in response to Pi-deficiency in plants, most of which focused on model plants or annual crops [16], [20], [50], [51]. In the present study, we focused on acclimation to Pi-deficiency in Masson pine using a transcriptomics approach based on a combination of RNA-seq and microarray experiments. We carefully designed the experiments to distinguish sets of genes that are involved in response to Pi-deficiency at different time points and at different levels of Pi-deficiency. A total of 1,396 differentially expressed transcripts were identified in P1 stress seedlings, and 943 were identified in P2 plants. A greater number of genes were therefore differentially expressed under conditions of lower Pi. Most of the differentially expressed genes fell into three major functional groups: metabolism, protein synthesis and stress response. To our knowledge, this is the first whole transcriptome analysis on Masson pine to address Pi-deficiency.

### Expression profiles of genes involved in the response to Pi-deficiency

A slow-growing plant will need less nutrients over a particular time period than a fast-growing individual, since the demand for nutrients is correlated with dry weight production [53]. No visible anthocyanin accumulated in the seedlings used in the current study, and no other growth differences were apparent in the aerial tissues during the duration of the experiments, in agreement with previous observations [37]. Since Masson pine is a relatively slow-growing plant, seedlings from four time periods (see Materials and methods) were chosen to analyze gene expression differences. The results showed that most differentially expressed transcripts were stably up- or down-regulated, suggesting that some functional genes are expressed continuously and efficiently in slow-growing plants.

### Alteration of lipid metabolism and membrane composition under Pi deficiency

The lipid composition of plant membranes can change drastically under conditions of Pi-deficiency, with an increase in non-phosphorus-containing lipids and a decrease in phospholipids [54]. Phosphate-deficient oat replaces a major portion of the plasma membrane phospholipids with the galactolipid digalactosyldiacylglycerol, and phospholipids are replaced by glycolipids in both the plasma membrane and tonoplast [55], [56]. Currently, an UDP-glycosytransferase (e.g., comp31295_c0_seq4, comp19255_c0_seq1, comp13888_c0_seq1) was up-regulated under P1 and P2 treatments, which indicated that the membrane lipid composition might have changed in response to low Pi stress.

### Expression of genes involved in Pi sensing and transport

All transcripts identified were differentially expressed under P1 and P2 conditions. Of particular interest, transcription of genes involved in Pi sensing and transport exhibited a >2-fold change in expression levels under P1 conditions, whereas these genes were practically unaffected by the P2 treatment. SPX domain-containing proteins are involved in Pi signaling and transport, and are essential for maintaining Pi homeostasis [5], [57]–[60]. Twenty SPX domain-containing proteins have been identified in *Arabidopsis*. One of the transcripts identified in P1 treated seedlings is an orthologue of AtSPX-MFS1 (At4g22990), which is a predicted transmembrane protein targeted to the vacuole that is weakly induced by Pi-deficiency [59]. However, it is difficult to draw any functional comparisons between SPX isoforms in Masson pine and *Arabidopsis* from the currently available data. The SPX domain-containing gene (comp23145_c2_seq1) identified herein was markedly up-regulated in P1 treatment, but no correlation between expression levels and phenotypic traits were observed. A total of five phosphate transporters were also up-regulated in P1 seedlings, and expression of these genes does appear to correlate with expression of the SPX domain-containing protein. The transcriptional changes identified in the P1 seedlings displayed a much higher magnitude and dynamic range than those identified in the P2 treatments. In addition to phosphate TPs, a potassium transporter was also up-regulated in P1, suggesting that the transport of phosphorus might be co-regulated and/or coordinated with potassium in this species, as documented previously in tomato [61]. Pi-deficiency (both P1 and P2) also increased the expression patterns of a number of ABC transporters, which have been shown to be induced under various abiotic stress conditions [62]–[64]. An anion exchanger presumably involved in Pi removal and absorption is one such protein [65], [66]. Our results also indicated an increased transport of sugars (comp14752_c0_seq1, comp324303_c0_seq1), amino acids (comp31360_c0_seq5, comp20063_c0_seq1), and organic acids (comp16177_c0_seq1), any of which could be involved in Pi mobilization and acquisition.

### Transcription factor expression was altered in response to Pi stress

The major TFs induced by Pi-deficiency (in both P1 and P2) belonged to the AP2, MYB, WRKY, and MADS-Box families. This is in contrast to previous studies from *Arabidopsis* in which the major TFs affected by Pi stress belonged to the *PHR*, *WRKY*, *ZAT*, and *bHLH* subfamilies. In *Arabidopsis*, *WRKY75* was up-regulated by Pi-deficiency stress, and in turn positively regulated Pi starvation-inducible genes and negatively regulated root development [67]. WRKY6 and WRKY42 are involved in the response to Pi-deficiency by regulating *PHOSPHATE1* (*PHO1*) expression [68], which plays a key role in Pi translocation from roots to shoots, aiding homeostasis [69]–[71]. In the present work, WRKY TFs were up-regulated in Masson pine under both P1 and P2 conditions (Table S5). The largest class of TFs induced by low Pi stress in our experiments was the MYB TF family. A total of 12 MYBs (10 in P1 and two in P2) were up-regulated by Pi-deficiency in Masson pine seedlings. One of these, *Phosphate Starvation Response1 (PHR1)*, was previously identified as a regulator involved in Pi-deficiency that activates a subset of genes including SPX domain-containing proteins by binding to the P1BS element in their promoter regions [59], [72]. Another, *AtMYB2*, regulates the plant response to Pi starvation by regulating the expression of the *miR399* gene in *Arabidopsis* [73]. Recently, Hernández et al. (2007) identified four TFs that were up-regulated in Pi-deficient common bean roots, three of which belonged to the MYB family [25]. Furthermore, a total of 33 MYBs were up-regulated in Pi deficient lupin leaves [16]. Therefore, MYB TF family proteins would be expected to play a role in the response to Pi-deficiency in Masson pine, which explains the results obtained. Plant MADS domain proteins were first identified as regulators of floral organ identity and had been found to control additional developmental processes such as flowering time, fruit dehiscence, meristem identity and root development [74]. Zhang and Forde (1998) identified a nitrate-inducible *Arabidopsis* gene (*ANR1*) that encodes a MADS-Box TF, and demonstrated that it controls nutrient-induced changes in root architecture [75]. Another MADS-Box gene (*CaJOINTLESS*) is involved in suppression of vegetative growth in shoot meristems in pepper [76]. Pi-deficiency can profoundly disturb root meristem maintenance [77], but it is still unknown whether MADS-Box genes are involved in acclimation to low Pi. In the current study, four MADS-Box transcripts were down-regulated in P1 seedlings and two were down-regulated in P2 treatment, suggesting that they may be involved in promoting meristem growth of Masson pine. The much higher root-to-shoot ratio (R/S) under Pi-deficiency conditions observed herein strongly indicated that down-regulation of MADS-Box TFs promoted root development in Masson pine to assist acclimation to low Pi conditions.

### Responses to oxidative stress

Metabolic pathways continuously generate ROS as by-products that inflict oxidative damage to cellular components and serve as critical signaling molecules in cell proliferation and survival [16], [78]. ROS homeostasis is usually maintained via regulation of antioxidant levels, but biotic and abiotic stresses can disturb the equilibrium between ROS formation and the antioxidant response, resulting in oxidative stress. The magnitude of Pi-deficiency has been directly correlated with the severity of oxidative stress [79], [80]. In this study, microarray data showed that glutathione s-transferase and glutathione peroxidase, which are both involved in cellular detoxification [80], [81], were up-regulated in Pi-deficient conditions, in agreement with previous studies [16], [82]. AO is an enzyme localized at the cell wall that uses oxygen to catalyze the oxidation of ascorbate to generate the unstable monodehydroascorbate (MDHA) radical, which rapidly disproportionates to yield dehydroascorbate (DHA) and ascorbate, and thus contributes to regulation of the ascorbate redox state. AO-overexpressing transgenic plants were found to exhibit a reduced capacity to up-regulate plant defenses against ROS [83]. Furthermore, suppression of AO expression conferred resistance to oxidative stress [84]. It appears that negligible expression of AO is a biological marker of severe oxidative stress in plants. In the current case, *AO* was down-regulated in P1 seedlings, which provides further evidence that severe Pi-deficiency induces severe oxidative stress. In our previous investigation, superoxide dismutase (SOD), peroxidase (POD) and catalase (CAT) activities were up-regulated in Masson pine under the conditions of Pi-deficiency (unpublished data), which further indicates that Pi-deficiency disturbs ROS homeostasis.

## Conclusion

To our knowledge, this work is the first report of a whole transcriptome analysis of Pi-deficiency in Masson pine, and firstly demonstrated the long-term transcriptomic changes that occur under different low-Pi growth conditions. A total of 1,396 transcripts were differentially expressed under severe Pi-deficiency (P1) and 943 transcripts were differentially expressed under moderate Pi-deficiency (P2). Stress-associated genes and transcription factors were particularly well represented, and some genes of unknown function were also up- or down-regulated. Most of the identified genes were consistent with previously reported studies on Pi-deficiency, but novel pathways were also identified. The available data suggested that the Pi-deficiency tolerance of Masson pine is a very complex physiological and biochemical process, in which many genes and multiple metabolism pathways were cumulatively implicated. This contribution was also significative to the development of genomic resources of Masson pine and other coniferous trees and should accelerate the progress of breeding programs and functional genomic studies.

## Supporting Information

Table S1 **Transcripts differentially expressed during different durations of Pi-deficiency stress experienced by P1 seedlings.** Values are normalized against the microarray data (P<0.05) and log 2 transformed. Where possible, annotations are included.
(XLS)

Table S2 **Transcripts differentially expressed during different durations of Pi-deficiency stress experienced by P2 seedlings.** Values are normalized against the microarray data (P<0.05) and log 2 transformed. Where possible, annotations are included.
(XLS)

Table S3 **Transcripts differentially expressed in P1 and P2.** 179 transcripts were calculated and normalized using GeneSpring GX. Where possible, annotations are included.
(XLS)

Table S4 **Transporter proteins differentially expressed under Pi-deficiency stress.**
(XLS)

Table S5 **Transcription factors differentially expressed under Pi-deficiency stress.**
(XLS)
